# Prediction of Antifungal Activity of Gemini Imidazolium Compounds

**DOI:** 10.1155/2015/392326

**Published:** 2015-04-15

**Authors:** Łukasz Pałkowski, Jerzy Błaszczyński, Andrzej Skrzypczak, Jan Błaszczak, Alicja Nowaczyk, Joanna Wróblewska, Sylwia Kożuszko, Eugenia Gospodarek, Roman Słowiński, Jerzy Krysiński

**Affiliations:** ^1^Department of Pharmaceutical Technology, Nicolaus Copernicus University, Jurasza 2, 85-094 Bydgoszcz, Poland; ^2^Institute of Computing Science, Poznań University of Technology, Piotrowo 2, 60-965 Poznań, Poland; ^3^Institute of Chemical Technology, Poznań University of Technology, Skłodowskiej-Curie 2, 60-965 Poznań, Poland; ^4^Department of Organic Chemistry, Nicolaus Copernicus University, Jurasza 2, 85-094 Bydgoszcz, Poland; ^5^Department of Microbiology, Nicolaus Copernicus University, Skłodowskiej-Curie 9, 85-094 Bydgoszcz, Poland; ^6^Systems Research Institute, Polish Academy of Sciences, Newelska 6, 01-447 Warsaw, Poland

## Abstract

The progress of antimicrobial therapy contributes to the development of strains of fungi resistant to antimicrobial drugs. Since cationic surfactants have been described as good antifungals, we present a SAR study of a novel homologous series of 140 bis-quaternary imidazolium chlorides and analyze them with respect to their biological activity against *Candida albicans* as one of the major opportunistic pathogens causing a wide spectrum of diseases in human beings. We characterize a set of features of these compounds, concerning their structure, molecular descriptors, and surface active properties. SAR study was conducted with the help of the Dominance-Based Rough Set Approach (DRSA), which involves identification of relevant features and relevant combinations of features being in strong relationship with a high antifungal activity of the compounds. The SAR study shows, moreover, that the antifungal activity is dependent on the type of substituents and their position at the chloride moiety, as well as on the surface active properties of the compounds. We also show that molecular descriptors MlogP, HOMO-LUMO gap, total structure connectivity index, and Wiener index may be useful in prediction of antifungal activity of new chemical compounds.

## 1. Introduction

In recent years the number of applications of quaternary ammonium compounds (QACs) has increased considerably. Gemini QACs are a group of cationic surfactants containing two head groups and two aliphatic chains linked by a spacer group.

Practical implementation of gemini QACs is a result of their surface active, antielectrostatic, and antimicrobial properties.

It has been demonstrated that gemini QACs exhibit properties superior to mono QACs, such as better solubility, higher adsorption efficiency, and better wetting and foaming [1–4]. Gemini QACs are more efficient in lowering surface tension and have much lower critical micelle concentration (CMC) [5]. Due to their higher surface activity they have excellent dispersion stabilization and soil clean-up properties [6, 7]. It has been also demonstrated that gemini QACs have good antifungal activity [8–10], which is higher than mono QACs [11, 12]. So it is worth developing new, more effective compounds, such as gemini QACs.

Because of the increasing resistance of microorganisms to commonly used disinfectants, the synthesis of new types of microbicides is a very important topic [13]. Formation of resistant strains of fungi is not as common as formation of resistant strains of bacteria [14]. Nevertheless, knowledge of properties of chemical compound, which influence the antifungal activity of gemini QACs, enables designing and synthesis of new, active chemical entities.

The main goal of our study was to investigate relationships between selected molecular parameters and features describing chemical structure and surface active properties and antifungal activity (described as MFC (minimal fungicidal concentration)). In MFC study* Candida albicans* ATCC 90028 strain was used. In structure-activity relationship study (SAR), modified method, based on a rough set theory, was employed.

*Candida albicans* is one of major opportunistic pathogens causing a wide spectrum of diseases in human beings. It can cause infections that range from superficial infections of the skin to life-threatening systemic infections [15]. Given the limited number of suitable and effective antifungal agents, together with increasing drug resistance of the pathogens, it is important that new classes of antifungals are discovered [16]. Moreover, better understanding of which features of chemical compounds decide high antifungal activity may provide further information useful for the improvement of antifungal action.

Data that describe the analyzed series of gemini imidazolium chlorides can be seen as classification data, where parameters characterizing structure and surface active properties, as well as molecular parameters, are condition attributes (independent variables) and antifungal activity is represented by class labels assigned to chlorides by a decision attribute (dependent variable). Structure-activity relationships can be discovered from these data by explaining the class assignment in terms of condition attributes. To this end, we applied the rough set concept [17], and its particular extension called Dominance-Based Rough Set Approach (DRSA) [18–21].

## 2. Materials and Methods

### 2.1. Gemini Imidazolium Chlorides

We analyzed 10 homologous series of gemini imidazolium chlorides with hydrophobic chain ranging from CH_3_ to C_16_H_33_ and with the length of spacer from C_2_ to C_12_. Synthesis, surface active properties, and antimicrobial activity of a part of 140 3,3′-(*α*,*ω*-dioxaalkyl)bis(1-alkylimidazolium) chlorides were described earlier [22]. Moreover, we determined molecular descriptors for synthetized structures. The antifungal activity was determined by the MFC values. The final stage of our study was an analysis of structure-activity relationships using DRSA [21].

### 2.2. Chemical Structure

**Figure 1:**
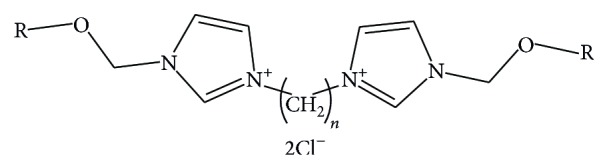
Chemical structure of analyzed compounds.

Chemical structure of chlorides was described by the following parameters (see Figure 1 and Table 1):

- *n*: number of carbon atoms in *n*-spacer,
- *R*: number of carbon atoms in *R*-substituent.

### 2.3. Surface Active Properties

Surface active properties of analyzed chlorides were described by the following parameters:

- CMC: critical micelle concentration (mol/L),
- *γ*CMC: value of surface tension at critical micelle concentration (mN/m),
- Γ × 10^6^ (*G*): value of surface excess (mol/m^2^),
- *A* × 10^−20^: molecular area of a single particle (m^2^),
- Δ*G*_ads_: free energy of adsorption of molecule (kJ/mol).

### 2.4. Molecular Parameters

We also considered molecular parameters of analyzed compounds, which were calculated with Dragon and Gaussian software. Molecular descriptor is the final result of a logic and mathematical procedure which transforms chemical information encoded within a symbolic representation of a molecule into a useful number or a result of a standardized experiment [23]. Those parameters were

- MLOGP: Moriguchi octanol-water partition coefficient,
- Balaban index (BI), Narumi topological index (NTI), total structure connectivity index (TSC), Wiener index (WI): numerical parameters characterizing compounds' topology,
- HOMO: highest occupied molecular orbital,
- LUMO: lowest unoccupied molecular orbital,
- HOMO-LUMO gap (HL gap): the energy difference between the HOMO and LUMO,
- dipole (dip): electric dipole moment,
- radius of gyration (ROG): the root mean square distance of the entities' parts from either its center of gravity or a given axis,
- molecular weight (MW) of compounds.

### 2.5. Antifungal Activity

*Candida albicans* ATCC 90028 microorganisms were used to evaluate antifungal activity of compounds by minimal fungicidal concentration (MFC). MFC determination method was presented in [22].

According to the value of MFC objects were sorted into three decision classes:

- class good: good antifungal properties: MFC ≤ 0.028 mM/L,
- class medium: medium antifungal properties: 0.028 < MFC < 0.1 mM/L,
- class weak: weak antifungal properties: MFC ≥ 0.1 mM/L.

Values of MFC for activity classes were determined on the basis of antimicrobial activity of benzalkonium chloride and didecyldimethylammonium chloride used as reference antifungals.

### 2.6. SAR Analysis Based on DRSA—Description of the Method

DRSA assumes that the value sets of condition attributes are ordered and monotonically dependent on the order of decision classes. DRSA proved to be an effective tool in analysis of classification data which are partially inconsistent [24, 25]. In the context of this study, inconsistency means that between a pair of chlorides the first one has not worse surface active and molecular properties than the other, although the first one is assigned to a worse class of antifungal activity than the other. The rough set analysis of consistent and inconsistent chlorides prepares the ground for induction of decision rules. The rules derived from data structured using the concept of the DRSA are monotonic, which means that they have the following syntax:

 “if at_*i*_(chloride) ≥ val_*i*_ and at_*j*_(chloride) ≥ val_*j*_ and ⋯ and at_*p*_(chloride) ≥ val_*p*_, then chloride is assigned to at least a given class,”
 “if at_*k*_(chloride) ≤ val_*k*_ and at_*l*_(chloride) ≤ val_*l*_ and ⋯ and at_*s*_(chloride) ≤ val_*s*_, then chloride is assigned to at most a given class,”

where at_*h*_ is an *h*th condition attribute and val_*h*_ is a threshold value of this attribute, which makes an elementary condition at_*h*_(chloride) ≥ val_*h*_ or at_*h*_(chloride) ≤ val_*h*_ composing a condition part of a rule indicating assignment of a chloride to at least (or at most) a given class (weak, medium, or good), respectively. In the above syntax of the rules, it is assumed that value sets of all condition attributes are numerical and ordered such that the greater the value, the more likely it is that the chloride has good antifungal activity; analogously, it is assumed that the smaller the value, the more likely it is that the chloride has weak antifungal activity. Attributes ordered in this way are called gain-type. Cost-type attributes have value sets ordered in the opposite direction, such that elementary conditions on these attributes have opposite relation signs. In case of gemini imidazolium chlorides data, it is not known* a priori* whether condition attributes are gain or cost attributes. Therefore, we proceeded as described in [26]: each original attribute is considered in two copies, with the first copy assumed to be gain-type and the second cost-type. The applied transformation of data is noninvasive; that is, it does not bias the relationships identified between condition attributes and the decision attribute. Then, an induction algorithm constructs decision rules involving elementary conditions on one or both copies of particular attributes. For example, for a rule indicating the assignment of a chloride to class good (at least good), the following elementary conditions concerning attribute at_*i*_ may appear:

- ↑at_*i*_(chloride) ≥ val_*i*1_,
- ↓at_*i*_(chloride) ≤ val_*i*2_,
- ↑at_*i*_(chloride) ≥ val_*i*1_ and ↓at_*i*_(chloride) ≤ val_*i*2_, which boils down to at_*i*_(chloride) ∈ [val_*i*1_, val_*i*2_,] if val_*i*1_ ≤ val_*i*2_,

where ↑at_*i*_ and ↓at_*i*_ are gain-type and cost-type copies of attribute at_*i*_, respectively. Note that this transformation of attributes allows global and local monotonic relationships to be discovered between condition attributes and class assignment. A monotonic relationship is global when it can be expressed by a single elementary condition concerning gain-type or cost-type attribute. Local monotonicity relationship requires conjunction of two elementary conditions of different types. In case of assignment of a chloride to class good we can have such a local monotonicity relationship; for example, when concentration of a surface active property is below a certain point, the greater the value the better the assignment, but after that point further increase may have a negative effect (i.e., the lower the value the better the assignment).

## 3. Results and Discussion

### 3.1. Information System

Information system is the basis of SAR analysis of the chemical compounds. It includes a set of objects (in rows) described by a set of attributes (in columns). The set of attributes is composed of condition and decision attributes. In our case, condition attributes describe surface active properties, molecular descriptors, and structure (the length on *n* spacer and the length of *R*-chain) of analyzed chlorides. The decision attribute concerns antifungal properties of bis-quaternary imidazolium chlorides represented by some limit values of MFC for* Candida albicans* ATCC 90028. A part of information system is presented in Table 2.

### 3.2. Decision Rules

Table 3 includes strong and relevant decision rules obtained for good and weak classes of chlorides presented in Table 2. These are rules selected from the set of all minimal decision rules induced from information table processed by DRSA.

We did not induce rules for class “medium” since these rules are not interesting from the viewpoint of SAR analysis (it is more important to know what are the features of chlorides with definitely good or weak antimicrobial properties). However, the presence of chlorides from the “medium” class is important in the rule induction process. The rules with conclusion “good” discriminate chlorides with “good” antimicrobial properties from those chlorides which have “medium” or “weak” properties (analogously for rules with conclusion “weak”).

The decision rules provide guidelines for synthesis of new compounds with better antifungal properties. The rules are characterized by various parameters, such as examples (i.e., number of objects covering a given rule), strength (i.e., the proportion of objects covered by premise that are also covered by conclusion), or confirmation (i.e., measure that is quantifying the degree to which premise provides evidence for conclusion).

In Table 3 only attributes that were present in decision rules are included.

Rules are characterized by their strength defined as a ratio of the number of chlorides matching the condition part of the rule to the total number of chlorides in the sample. Sets of decision rules, which are essential for the analysis presented in this work, were induced from gemini imidazolium chlorides data, which were collected in an information system. A part of the system can be seen in Table 2. These data were transformed as described above and structured according to the DRSA. The induction algorithm that was applied to construct rules is called VC-DomLEM [27]. The algorithm was implemented as a part of software package called jMAF (http://idss.cs.put.poznan.pl/site/139.html), based on the java Rough Set (jRS) library. The sets of induced rules were used to construct component classifiers in variable consistency bagging [28, 29]. Variable consistency bagging (VC-bagging) [29] was applied to increase the accuracy of results produced by VC-DomLEM.

Both rule relevance and relevance of attribute, which are present in condition part of rules, were estimated by measuring Bayesian confirmation, as described in [30]. In this process, decision rules were constructed repetitively on bootstrap samples and tested with chlorides that were not included in the samples.

In the “good” class of antifungal activity strong rules, supported by a large number of objects, were obtained. The most interesting rules are characterized by high confirmation measures. In decision rules covering chlorides with good activity against* Candida albicans*, chlorides with *n*-spacer longer or equal to 6 atoms of carbon predominate. We can also observe that optimal length of *R*-substituent is from 7 to 11 carbon atoms in a chain. Moreover, those rules emphasize that *γ*CMC is important from the point of view of assigning new compounds into a good class of activity. As it was mentioned before, we included molecular descriptors into our SAR analysis. Results are as follows: Moriguchi octanol-water partition should be in the range [3.836; 6.94], the energy difference between the HOMO and LUMO should be less than or equal to −0.17314, Balaban index should be greater than or equal to 1.242, Narumi topological index should be greater than or equal to 21, and total structure connectivity index should be less than or equal to 0.218.

When we consider assigning new chlorides into weak decision class, the length of *n*-spacer in compound's moiety should be shorter or equal to 6 atoms of carbon. We can also observe that values of surface tension at critical micelle concentration greater or equal to 50.1, values of surface excess greater or equal to 2.48, and values of free energy of adsorption of molecule less than or equal to 23.2 are important when considering weak activity against* Candida albicans* strains. Decision rules for weak class of chlorides include only one molecular descriptor, Moriguchi octanol-water partition coefficient, in contrast to good activity class, which included all molecular descriptors, besides Wiener index.

### 3.3. Attribute Relevance

**Figure 2:**
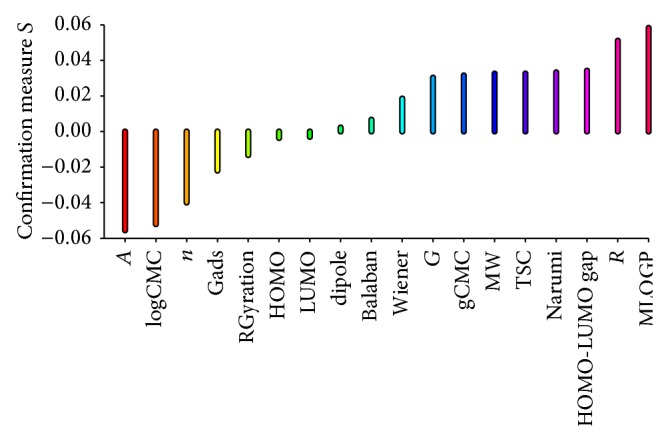
Predictive confirmation of attributes for class good.

**Figure 3:**
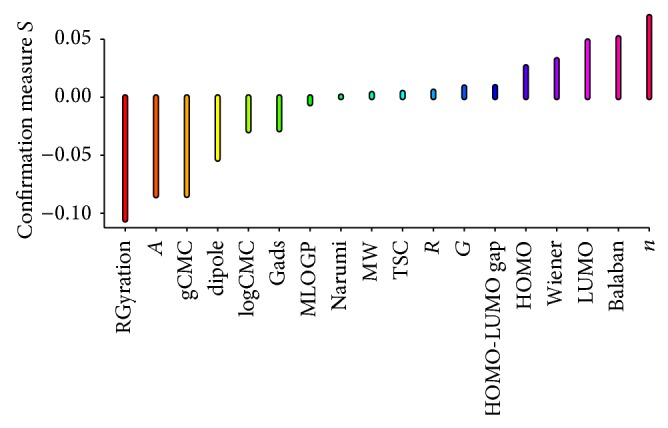
Predictive confirmation of attributes for class weak.

Results of estimation of predictive confirmation of all attributes (structure, surface active, and molecular ones) in rules induced for class good and weak are presented in Figures 2 and 3.

Let us interpret a rule as a consequence relation “*if E*,* then H*,” where *E* denotes rule premise and *H* rule conclusion. For rule relevance, the Bayesian confirmation measure quantifies the contribution of rule premise *E* to correct classification of unseen individuals. Many Bayesian confirmation measures have been described in the literature, of which we used the measure *s*(*H*, *E*). This approach allows clear interpretation in terms of a difference of conditional probabilities involving *H* and* E*; that is, *s*(*H*, *E*) = *Pr*⁡(*H*∣*E*) − *Pr*⁡(*H*∣¬*E*), where probability *Pr*⁡(·) is estimated from the test samples of chlorides. For the relevance of single attributes, the Bayesian confirmation measure quantifies the degree to which the presence of attribute at_*i*_ in premise *E*, denoted by at_*i*_⊳*E*, provides evidence for or against conclusion *H* of the rule. Here, we used again measure *s*(*H*, at_*i*_⊳*E*), which, in this case, is defined as follows: *s*(*H*, at_*i*_⊳*E*) = *Pr*⁡(*H*∣at_*i*_⊳*E*) − *Pr*⁡(*H*∣at_*i*_¬⊳*E*). Consequently, attributes present in the premise of a rule that assigns chlorides correctly or attributes absent from the condition part of a rule that assigns chlorides incorrectly are considered more relevant.

We can observe that attributes Moriguchi octanol-water partition coefficient, the length of *R* substituent, and HOMO-LUMO gap are the most relevant when the good class of activity is considered. On the other hand, the most relevant attributes for weak decision class are the length of *n*-spacer, Balaban index, and LUMO parameter. These results show that all three types of parameters: structure, surface active, and molecular might be helpful in assigning new chemical entities to a specific class of antifungal activity.

Chemical structure of gemini surfactants influences not only their surface properties, but also their antimicrobial activity. It has been widely accepted that optimal antimicrobial activity can be obtained from 10 to 18 atoms of carbon in an aliphatic chain, with an optimum of 12 to 16 atoms of carbon, depending on a bacterial strain [31]. An elongation of the hydrophobic chain increases antimicrobial activity, but only to a given limit, after which, activity decreases. It was also observed that the lowest MFC values are specific for medium-length hydrophobic substituents attached to a quaternary atom of nitrogen [32]. Similar observations can be found in [33]. Specific properties of gemini compounds, with the above mentioned length of hydrophobic substituents, are related to their ability to form and coexist with small spherical micelles and large aggregates. Below this range only micelles are found, while above this range only aggregates are observed [34].

In this paper, it was found that good antifungal activity for a group of analyzed gemini chlorides is related to *n*-spacer equal to or longer than 6 atoms of carbon. Moreover, we discovered more features being in a strong relationship with a good antifungal activity, regarding* Candida albicans* strains. Those are not only the length of substituents in a moiety but also logCMC and *γ*CMC, Moriguchi octanol-water partition coefficient, the energy difference between the HOMO and LUMO, Balaban index, Narumi topological index, and total structure connectivity index. Those parameters should be taken into consideration when one will plan synthesis of new gemini chloride with a high anti-*Candida albicans* activity.

### 3.4. Results of Stratified Cross-Validation

The model constructed by VC-bagging with VC-DomLEM component classifiers showed good classification performance in 5-fold stratified cross-validation, which was repeated 100 times for a better reproducibility of results. First, we considered accuracy of distinction between chlorides that have good and not good (i.e., medium or weak) antifungal activity properties. In this case, on the average, 77.3% of chlorides were correctly classified (81.9% were correctly classified as having good properties, and 70.7% were correctly classified as having not good properties). Second, we checked distinction between chlorides having weak and not weak (i.e., medium or good) antifungal activity properties. On the average, 86.2% of chlorides were correctly classified in this case (80.9% were correctly classified as having weak properties and 88.1% were correctly classified as having not weak properties).

## 4. Conclusions

Decision rules presented in this study show that number of carbon atoms in *n*-spacer, number of carbon atoms in *R*-substituent, MlogP, HOMO-LUMO gap, total structure connectivity index, and Wiener index have the most influence on the increase of antifungal activity of 3,3′-(*α*,*ω*-dioxaalkyl)bis(1-alkylimidazolium) chlorides. On the other hand, number of carbon atoms in *n*-spacer, value of surface excess, and Wiener index affected decreasing of antifungal activity of studied gemini imidazolium chlorides. Obtained results are directions for synthesis of new active molecules of gemini imidazolium chlorides possessing strong antifungal action. DRSA is a valuable tool to conduct SAR analysis of chemical compounds.

## Figures and Tables

**Table 1:** Numerical coding of the structure of analyzed chlorides.

Code	Condition attributes	
	*n*-spacer	*R*-substituent
1		CH_3_
2	C_2_H_5_	C_2_H_5_
3	C_3_H_7_	C_3_H_7_
4	C_4_H_9_	C_4_H_9_
5	C_5_H_11_	C_5_H_11_
6	C_6_H_13_	C_6_H_13_
7	C_7_H_15_	C_7_H_15_
8	C_8_H_17_	C_8_H_17_
9	C_9_H_19_	C_9_H_19_
10	C_10_H_21_	C_10_H_21_
11		C_11_H_23_
12	C_12_H_25_	C_12_H_25_
14		C_14_H_29_
16		C_16_H_33_

**Table 2:** A part of information system (10 from 140 objects).

Number	*n*	*R*	lgCMC	gCMC	*G*	*A*	*G*_ads_	MLOGP	BI	NTI	WI	MW	HOMO	LUMO	HL gap	dip	ROG	TSC	MIC [mM/L]	Class
1	2	1	2,15	61,9	2,75	52	20,2	0,175	1,397	12,712	5,275	252,36	−0,38777	−0,19852	−0,18925	1,646	4,908	0,28	16,937	Weak
2	2	2	2,23	60,1	2,71	54	20,8	0,711	1,407	14,099	5,768	280,42	−0,38416	−0,19108	−0,19308	0,103	5,294	0,266	3,558	Weak
3	2	3	2,38	59,8	2,69	56	21,3	1,216	1,405	15,485	6,307	308,48	−0,38269	−0,18871	−0,19398	2,314	5,804	0,254	3,295	Weak
4	2	4	2,41	57,4	2,65	58	21,7	1,697	1,397	16,871	6,877	336,54	−0,38194	−0,18751	−0,19443	5,474	6,246	0,243	1,634	Weak
5	2	5	2,49	55,5	2,61	60	22,3	2,157	1,386	18,257	7,468	364,6	−0,38150	−0,18679	−0,19471	8,628	6,777	0,234	0,712	Weak
6	2	6	2,58	53,4	2,57	62	22,7	2,599	1,373	19,644	8,074	392,66	−0,38544	−0,18641	−0,19903	12,501	7,25	0,226	0,086	Medium
7	2	7	2,65	51,2	2,53	64	23,5	3,025	1,359	21,03	8,692	420,72	−0,38109	−0,18618	−0,19491	16,201	7,791	0,218	0,020	Good
8	2	8	2,72	48,9	2,49	66	23,9	4,349	1,346	22,416	9,319	448,78	−0,36560	−0,18605	−0,17955	20,472	8,282	0,211	0,005	Good
9	2	9	2,81	47,5	2,45	68	24,3	4,748	1,333	23,803	9,952	476,84	−0,35218	−0,18590	−0,16628	24,499	8,831	0,205	0,002	Good
10	2	10	2,92	45,3	2,41	70	24,8	5,136	1,32	25,189	10,59	504,9	−0,34105	−0,18584	−0,15521	29,011	9,333	0,199	0,017	Good

**Table 3:** Decision rules.

Number	Condition attributes														Examples	Strength	Confirmation measure *s *
	*n*	*R*	−log⁡CMC	*γ*CMC	Γ · 10^6^	A · 10^20^	Δ*G*_ads_	*M*log⁡*P*	MW	WI	HLgap	BI	TSC	NTI			
Decision class good																	
1		≤10											≤0.218		54	0.3857	0.7500
2		≤11						≥3.836							49	0.3500	0.7005
3											≤−0.17317			≥21.03	42	0.3000	0.8000
4											≤−0.15191		≤0.199		42	0.3000	0.7142
5		≤11											≤0.199		39	0.2785	0.7058
6	≥6								≥406.69		≤−0.17314				37	0.2642	0.6315
7	≥7								≥378.63			≥1.242			34	0.2428	0.6315
8	≥7			≤48.3								≥1.242			31	0.2214	0.6315
9		≥7					≥27.7	≤6.94							29	0.2071	0.6666
10		[7; 11]					≥26.2								29	0.2071	0.6666

Decision class weak																	
11	≤6				≥2.48										26	0.1857	0.9523
12	≤6				≥2.52										25	0.1785	0.9523
13	≤6							≤2.814							24	0.1714	0.9523
14	≤6				≥2.53										24	0.1714	0.9523
15	≤6		≤2.54												23	0.1642	0.9523
16	≤6				≥2.56										22	0.1571	0.9523
17	≤5			≥50.1											22	0.1571	0.9523
18	≤5				≥2.52										21	0.1500	0.9545
19	≤5			≥52.1											20	0.1428	0.9523
20	≤5						≤23.2								20	0.1428	0.9523
